# Congestive heart failure associated with itraconazole in a patient with paracoccidioidomycosis

**DOI:** 10.1016/j.bjid.2024.103868

**Published:** 2024-09-11

**Authors:** Hugo Haran Souza Andrade, Isabel Cunha Santos, Roger Lopes Batista, Mario León Silva-Vergara

**Affiliations:** Universidade Federal do Triângulo Mineiro, Departamento de Medicina Interna, Unidade de Doenças Infecciosas, Uberaba, MG, Brazil

**Keywords:** Paracoccidioidomycosis, Itraconazole, Congestive heart failure, Cardiotoxicity

## Abstract

Itraconazole (ITZ) is widely prescribed for the treatment of mycosis such as Paracoccidioidomycosis (PCM). However, it's related to toxicity and serious adverse events, such as Congestive Heart Failure (CHF). The objective is to describe a patient with PCM and CHF secondary to ITZ. Male, 50-years old, was diagnosed with chronic adult PCM and started ITZ 200 mg 12/12 h. After 2-months, acute CHF began without previous-heart disease. The electrocardiogram showed changes in ventricular repolarization and left anterior superior divisional block. Echocardiogram: slight reduction in left ventricular systolic function and ejection fraction of 51%. ITZ was replaced by trimethoprim-sulfamethoxazole. After a week, there was remission of symptoms. Despite thousands of patients around the world received ITZ, few cases of CHF were reported. It's dose dependent and improves when the drug is discontinuing. ITZ has negative inotropic effect and probably causes mitochondrial dysfunction. However, the intrinsic mechanisms are not yet completely understood.

## Introduction

Paracoccidioidomycosis (PCM) is one of the most prevalent endemic mycoses in Latin America where there are an estimated 10 million infected individuals. It mainly affects male patients in their productive age of life and is acquired through inhalation of fungal infections propagules during activities in rural areas. Most infected individuals remain asymptomatic and a minimal and unknown portion of them may develop the disease throughout their lives. The PCM frequency rate is unknown due to it is not notified in public health systems of most endemic countries.^1^

The chronic form of PCM represents 80% of cases, affects the lungs, mucous membranes, skin, and eventually other organs. The acute/subacute form presents with central and peripheral lymphadenopathy, fever, significant weight loss and severe impairment of the general condition. Skin and long bones can also be affected.^2^

The diagnosis of PCM is based on direct examination of body secretions with KOH and histopathology of tissue fragments. Serological and molecular techniques are more restricted to reference centers. PCM treatment can be carried out with amphotericin B in more severe cases during the induction phase followed by maintenance phase with oral ITZ for 9 to 18 months.^3^

ITZ has high efficacy and generally good tolerance and is widely used to treat superficial, subcutaneous and systemic mycosis. After approval in 1992 for use in humans, serious reports of toxicity associated with its use emerged, mainly Congestive Heart Failure (CHF). Thus, in 2001 after evaluating 58 reported cases, the Food and Drug Administration (FDA) issued a warning about this risk, and it was included in the drug's package insert. Since then, dozens of cases have been reported in the literature, even patients without risk factors for CHF.^4^^,^^5^

The mechanism of Cardiotoxicity (CT) due to ITZ is not yet well understood. It's believed to have a negative inotropic effect and cause mitochondrial dysfunction.^6^, ^7^, ^8^ The review of cases of CHF associated with ITZ shows that this may or may not lead to a reduction in ejection fraction. The average time until adverse reactions appear is 4 weeks and the risk of CT increases when the dose is greater than 400 mg/day. Reducing or stopping the medication results in improvement or reversal of congestive symptoms.^9^

The objective of this report is to describe the case of a patient with chronic PCM who started treatment with ITZ and developed CHF that improved after stopping the medicament.

## Case report

Male patient, 50 years old, Brazilian, black, agricultural machinery operator and from Santa Juliana, Minas Gerais, Brazil. For 7 months, he has had a dry cough and episodes of hemoptysis, thoracic pain and unquantified weight loss, in addition to dyspnea on medium exertion. The patient has a previous history of arterial hypertension under treatment and morbid obesity.

On physical examination, chest expansion was decreased bilaterally and there were fine rales in the left lung base. Chest X-Ray showed bilateral opacities and cardiac area size of 52%. Chest computed tomography showed consolidation in the lower lobe of the left lung and scattered nodules with cavitations in both lungs. In addition, lymphadenopathy was seen mediastinal and hilar on the left and scattered areas of pulmonary opacity. The polymerase chain reaction for tuberculosis was negative. Routine laboratory tests showed no notable changes. Electrocardiogram (ECG) showed sinus rhythm. The histopathology of a lung fragment obtained through by fiberoptic bronchoscopy carried out four months after the first consultation had an inconclusive result. The new fibrobronchoscopic examination showed a vegetative lesion in the trachea and the biopsy of the fragment obtained showed chronic granulomatous inflammation associated with fungi morphologically similar to *Paracoccidiodes* spp.

Once the diagnosis of chronic adult PCM was confirmed, ITZ produced by NovaMed NC Farma, 200 mg BID was prescribed and an outpatient return was scheduled in 30 days. In the first control, the patient reported significant improvement in cough, hemoptysis and dyspnea, but reported the onset of bilateral edema in the lower limbs. In the subsequent control, four months after diagnosis the edema was generalized and more intense. Furthermore, he complained orthopnea and paroxysmal nocturnal dyspnea. On physical examination, there were clear signs of pulmonary congestion, with presence of diffuse rales. Given these findings, the hypothesis of CHF secondary to ITZ was considered.

The patient was hospitalized, prescribed anticongestive therapy, and antihypertensive treatment was optimized. ITZ was replaced by trimethoprim-sulfamethoxazole to continue PCM treatment. The ECG showed a left shifted QRS complex, normal PR and QTc intervals, left anterosuperior divisional block and nonspecific changes in ventricular repolarization on the lateral and inferior sides. The echocardiogram showed a slight reduction in ejection fraction (51%). After 7 days of substantial improvement of his symptoms, he was discharged from hospital in good general condition and without signs and symptoms of CHF. In outpatient follow-up consultations and to this day the patient remains asymptomatic and undergoing treatment for PCM.

## Discussion

Since the beginning of the 1980s, ITZ began to be clinically evaluated for the treatment of superficial and systemic mycoses, such as paracoccidioidomycosis, aspergillosis and histoplasmosis, among others. At the time, low toxicity rates and greater efficacy were described in relation to other available azoles.^10^^,^^11^

Between 1992 and 2001, the FDA received 58 reports of possible CHF cases related to the drug. At the time, the possibility of this event was included in the leaflet, in addition to guidance on this contraindication in patients with previous heart disease or with important risk factors for CHF. Furthermore, no cases of CHF in association with other azole derivatives have been described, which allows to rule out that it is a class effect.^4^

In subsequent decades, some series of cases and isolated reports of CHF associated with ITZ were described (Table 1). In this review, more than hundred cases were identified, the majority aged 60 years or over, in which ITZ was used to treat various systemic mycosis such as histoplasmosis, aspergillosis and cryptococcosis, among others. The doses used were 400 mg/day or more in most cases. The time between the start of treatment and the appearance of toxicity symptoms varied from few days to weeks. The predominant symptomatology was CHF. The approach in most cases was to suspend the medication, with complete resolution of the condition in most cases (Table 1).

**Table 1:** Update of the main case reports of cardiotoxicity associated with itraconazole.

Reference	Patients number	Age/ years	Gender	Clinical indication	Daily dose/mg	Elapsed time to ITZ toxicity (weeks)	Clinical features of cardiotoxicity	Clinical decision	Outcome
^4^	58	(15‒86)	M: 9/55	Onychomycosis: 26/52	100‒800	1.4 (1‒30)	NA	NA	NA
			F: 6/55	Systemic mycosis: 15/52					
				Others 11/52					
^5^	1	38	M	Aspergillosis	400	<1	CHF	SD	CR
^9^	31	(56‒70)	M: 17	Histoplasmosis: 15	400 (23)	4 (0.3‒104)	CHF:13 Hypertension: 8	UD: 2	CR: 17
			F: 14	Aspergillosis: 2	200 (2)		Edema: 23	SD: 23	PR: 9
				Coccidioidomycosis: 3	600 (2)		Pericardial effusion: 1	DD: 6	Unchanged: 4
				Blastomycosis: 5	800 (1)		Others: 8		NA: 1
				Fungal prophylaxis: 1	NA (3)				
				Cryptococcosis: 1					
				Others:4					
^13^	1	59	M	Coccidioidomycosis	600	4	Hypertension	SD	CR
^6^	1	58	M	Aspergillosis	400	NA	CHF	SD	CR
	1	71	M	Aspergillosis	400	NA	CHF	SD	CR
	1	55	M	Aspergillosis	400	NA	Hypertension	UD	CR
	1	60	M	Aspergillosis	400	NA	Hypertension	UD	Death
^7^	1	31	M	Blastomycosis	NA	24	CHF	SD	Death
	1	64	M	Histoplasmosis	NA	12	CHF	SD	PR
^8^	1	76	M	Aspergillosis	400	2	CHF	SD	CR
^14^	1	60	F	Onychomycosis	400	<1	CHF	SD	CR
^15^	1	60	M	Dermatomycosis	200	6	CHF	SD	CR
^16^	1	66	M	Blastomycosis	200	8	CHF	SD	CR
^17^	1	75	M	Histoplasmosis	600	NA	Edema, crackles, orthopnea, jugular turgidity.	SD	CR
^18^	1	68	M	Aspergillosis	200	NA	Hypertension	SD	CR
^19^	1	22	M	Histoplasmosis	600	<1	CHF	NA	Death

M, Masculine; F, Feminine; NA, Not Available; UD, Unchanged Dose; SD, Suspended Dose; DD, Decreased Dose; CR, Complete Resolution; PR, Partial Resolution.

The patient reported here began treatment for chronic PCM with 400 mg/day of ITZ to due to his morbid obesity. Most cases with this disease use 200 mg/day as recommended by consensus.^3^ The patient had a normal ECG and had no symptoms or history of CHF or any other heart disease other than controlled arterial hypertension, despite the slight increase in the cardiac area seen at chest X-Ray.

The absence of cardiovascular risk factors or another underlying cardiac disease that could explain CHF and the chronological relationship between the start of the medication and the appearance of signs and symptoms of CHF, makes it possible to establish a causal relationship. This fact is reinforced by the score of 7 on the Naranjo score, which places the relationship with toxicity as probable when values are between 5 and 8, which consider among other criteria, the discontinuation of the medication accompanied by complete resolution of symptoms. Naranjo's score, which allows the relationship between ITZ and cardiac toxicity to be established with great accuracy. This method establishes a reliability coefficient of 0.92 for the intraclass correlation that can discriminate adverse reactions of different probabilities.^12^

The intrinsic mechanisms that induce cardiac toxicity are not yet completed understood. ITZ promotes a negative inotropic myocardial effect and probable mitochondrial dysfunction. Furthermore, it can lead to a decrease in heart rate, coronary flow, prolongation of the PR interval and QRS complex. Additionally, it has been described that ITZ can interfere with the biosynthesis pathway of steroid hormones by inhibiting the 11β-hidroxysteroid enzyme dehydrogenase type 2, which can induce high blood pressure and cause fluid and electrolyte disorders such as hypokalemia.^13^

After the clinical experience of treating several hundred cases of patients with paracoccidioidomycosis and other systemic mycoses at the institution, the reported case is the second observed in several decades. The first was from another young female patient with severe PCM who required conventional amphotericin B. A few days after starting this medication, she developed severe myocarditis which led to its discontinuation. With the clinical improvement of the myocarditis, ITZ 200 mg/day was started but within few days, the patient developed CHF. For this reason, this medication was suspended and treatment with trimethoprim-sulfamethoxazole continued.

When considering the inestimable number of patients who received ITZ for the treatment of various mycoses over the decades, the incidence of this toxicity appears to be low or little reported or published. However, cardiac evaluation for patients starting treatment with ITZ could be suggested as there are reports of cases of CHF even in patients with normal cardiac function.^5^

## Ethical considerations

A free and informed consent form was signed by the patient and the report was submitted to the ethical board of the teaching Hospital.

## Funding

This research did not receive any specific grant from funding agencies in the public, commercial, or not-for-profit sectors.

## Conflicts of interest

The authors declare no conflicts of interest.
